# Growth Patterns in Early Childhood and Final Attained Stature: Data from Five Birth Cohorts from Low- and Middle-Income Countries

**DOI:** 10.1002/ajhb.20998

**Published:** 2009-10-23

**Authors:** Aryeh D Stein, Meng Wang, Reynaldo Martorell, Shane A Norris, Linda S Adair, Isabelita Bas, Harshpal Singh Sachdev, Santosh K Bhargava, Caroline HD Fall, Denise P Gigante, Cesar G Victora, on behalf of the cohorts group

**Affiliations:** 1 Hubert Department of Global Health, Rollins School of Public Health, Emory UniversityAtlanta, Georgia; 2 Birth to Twenty Research Programme, University of the WitwatersrandJohannesburg, South Africa; 3 Department of Paediatrics, University of CambridgeCambridge, United Kingdom; 4 Department of Nutrition, Gillings School of Global Public Health, University of North Carolina at Chapel HillChapel Hill, North Carolina; 5 Office of Population Studies Foundation, University of San CarlosCebu, Philippines; 6 Department of Pediatrics, Sitaram Bhartia Institute of Science and ResearchNew Delhi, India; 7 Department of Pediatrics, S.L. Jain HospitalDelhi, India; 8 MRC Epidemiology Resource Centre, University of SouthamptonSouthampton, United Kingdom; 9 Universidade Federal de PelotasPelotas, Brazil

## Abstract

Growth failure is cumulative, and short stature is associated with multiple indices of reduced human capital. Few studies have been able to address in a single analysis both consideration of the timing of growth failure and comparison across populations. We analyzed data from birth cohorts in Brazil, Guatemala, India, the Philippines, and South Africa (*n* = 4,659). We used data on length at birth (available for three of the five cohorts), 12 mo, 24 mo, and mid-childhood to construct cohort- and sex- specific conditional length measures. We modeled adult height as a function of conditional length in childhood. The five cohorts experienced varying degrees of growth failure. As adults, the Brazil sample was 0.35 ± 0.89 standard deviations (SD) below the World Health Organization reference, while adult Guatemalans were 1.91 ± 0.87 SD below the reference. All five cohorts experienced a nadir in height for age *Z*-score at 24 mo. Birth length (in the three cohorts with this variable), and conditional length at 12 mo (in all five cohorts) were the most strongly associated with adult height. Growth in the periods 12–24 mo and 24 mo to mid-childhood showed inconsistent patterns across tertiles of adult height. Despite variation in the magnitude of cumulative growth failure across cohorts, the five cohorts show highly consistent age-specific associations with adult stature. Growth failure prior to age 12 mo was most strongly associated with adult stature. These consistencies speak to the importance of interventions to address intrauterine growth failure and growth failure in the first 12 mo of life. Am. J. Hum. Biol. 2010. © 2009 Wiley-Liss, Inc.

There is marked variability, both within and among countries, in the extent and timing of growth failure (Shrimpton et al., 2001). Long-term reductions in the prevalence of stunting have been reported in several settings, including Europe (Cole, 2000), Guatemala (Stein et al., 2009), Brazil (Monteiro et al., 2009), and China (Svedberg, 2006). Nevertheless, childhood growth retardation remains an important public health problem in lower-income countries and among the poorer strata of middle-income countries (Black et al., 2008).

In the aggregate, growth retardation in childhood leads to shorter attained height in adulthood. Shorter height is associated with reduced employment opportunities, lower wages, and other measures of human capital (Carba et al., 2009; Curi and Menezes-Filho, 2008; Thomas and Frankenberg, 2002), and severe stunting in women is associated with adverse reproductive outcomes (WHO, 1995). Prevention of growth retardation would therefore have large returns. It is generally presumed that interventions to reduce stunting need to be applied during (or even before) pregnancy and in the first 2–3 years of postnatal life (Heckman, 2006; Martorell et al., 1994), but there is some evidence of “catch-up" later in childhood (Eckhardt et al., 2005).

To address the question of the relative importance of early versus later childhood growth to adult stature, longitudinal data and statistical approaches that are able to identify and differentiate population and individual growth trajectories are needed. This article builds upon the COHORTS (Consortium on Health-Orientated Research in Transitional Societies) collaboration, a network of five birth cohorts with repeated measures of stature in childhood and follow-up at least through late adolescence. Our objective was to describe commonalities and differences among the cohorts in the patterns of child growth and the relation of these patterns to attained height. Of particular interest is the identification of periods of child growth that might be critical in determining final height.

## METHODS

### Study populations

We use data from five cohorts in low- and middle-income countries including: The 1982 Pelotas Birth Cohort (Brazil) (Victora and Barros, 2006), the Institute of Nutrition of Central America and Panama Nutrition Trial Cohort (Guatemala) (Stein et al., 2008), the New Delhi Birth Cohort (India) (Sachdev et al., 2005); the Cebu Longitudinal Health and Nutrition Survey (Philippines) (Adair, 2007), and the Birth to Twenty (South Africa) Cohort (Richter et al., 2007) (Table 1.) All field activities were reviewed and approved by an appropriate ethics committee or Institutional Review Board and all participants (or their parents, as appropriate) provided informed consent for all measures reported.

**TABLE 1:** Selected characteristics of the study cohorts

Cohort name	Design	Cohort inception year and initial sample *(N)*	Last follow-up visit year and number examined *(N)*	Initial cohort design	Ages at which length/height data were collected
Pelotas, Brazil	Prospective cohort	1982; 5,914	2004–2005; 4,297	Children born in the city's maternity hospital (>99% of all births) in 1982. All social classes included.	12 mo (25% sample), 24 mo, 48 mo, 18 years, 23 years
INTCS, Guatemala	Community trial	1969–1977; 2,393	2004; 1,571	Intervention trial of a high-energy and protein supplement in women, and children <7 years in 1969 and born during 1969–1977 in four villages.	15 d, 3, 6, 9, 12, 15, 18, 21, 24, 27, 30, 33, 36, 42, 48, 54, 60, 66, 72 mo; adolescence (in 1988); adulthood
New Delhi, India	Prospective cohort	1969–1972; 8,181	1998–2002; 1,583	Babies born to an identified population of married women living in a defined area of Delhi. Primarily middle-class sample.	Every 6 mo from birth to 21 years, adulthood
CLHNS, Philippines	Prospective cohort	1983–1984; 3,080	2005; 2,032	Pregnant women living in 33 randomly selected neighborhoods; 75% urban. All social classes included.	Birth, every 2 mo through 24 mo, 102 mo, adolescence, adulthood
Birth-to-twenty, South Africa	Prospective cohort	1990; 3,273	2008; 2,225	Babies born to pregnant women living in a defined urban geographical area. Predominantly poor, black sample.	6, 12, 24, 60, 132 mo, adolescence

Infant and child anthropometry. In each study, length or height was measured by the research teams using study-specific, but consistent, methodologies. Length (cm) was measured at birth in India, within 6 days of birth in Philippines, and at 15 days of birth in Guatemala. Birth length was not available for Brazil or South Africa. The studies varied in the timing and frequency of measurement (Table 1). In all studies except Guatemala, supine length was measured until 24 mo and standing height from age 24 mo. In Guatemala, supine length was measured through the age of 7 years, and for the present analysis we converted supine lengths to standing heights by subtracting 1.0 cm from lengths obtained at ages 24 mo and older.

For the present analysis, we used data for length at birth (Guatemala, India, Philippines) and 12 months (all 5 cohorts) and height at 24 months (all 5 cohorts) and at a point that we henceforth refer to as mid-childhood, namely 48 months (Brazil, Guatemala, India), 60 months (South Africa), and 102 months (Philippines). The India data were contributed to the pooled data set as values interpolated to exact ages of 12, 24, and 48 months using individual growth curves. All length and height measures were converted to *Z*-scores by comparing them to the 2006 World Health Organization (WHO) growth standards, which were generated from six cohorts of breast-fed infants and children free from economic constraints on growth (WHO, 2006) or the 2007 reference curves, which extend the age range through adolescence (de Onis et al., 2007) and were derived in part from the US National Center for Health Statistics reference curves. Use of *Z*-scores provides a common reference against which cohort-specific growth can be assessed.

### Anthropometry at follow-up

Height was measured by the research teams at the age of 15 years in South Africa, and in adulthood for the other sites. Height at follow-up was measured using a stadiometer in all studies. For simplicity, these measures are all referred to as adult measures. We converted adult heights to *Z*-scores using the WHO curves for adolescents (de Onis et al., 2007), assuming that height is unchanged from the age of 19 years.

### Statistical methods

Analytic sample. Our main analysis sample (*n* = 4,659) includes participants from each site who had height measured during the most recent follow-up, and length or height measured at 12, 24 months, and during mid-childhood. In some analyses, we further restricted the sample to those with birth length.

We computed means and SD of height for age *Z*-score (HAZ) at each age for each site. We categorized individuals by site- and sex-specific tertiles of adult height, and plotted the HAZ at each age within these groupings. We compared height at 24 mo and growth age 24 mo to adulthood across the five cohorts and against the 50th percentile of the reference distribution.

To eliminate some of the statistical problems associated with modeling highly correlated length measures, we use “conditional length” variables to represent length (or height) at a given age, independent of length at earlier ages (Adair et al., 2009). Conditional length is the residual derived by regressing length at each age on length at birth (length at 12 mo for models without birth length measures) and at all prior ages (all expressed in their original metrics), and thus represents a child's deviation from his or her expected size given the pattern of growth in that population. By design, there is no correlation among the baseline value (birth or 12 mo, as appropriate) and subsequent conditional size measures. Models were site- and sex-specific, included exact age at measurement, and accounted for nonlinear relationships by including squared prior length. We standardized the residuals to allow comparison of the size of coefficients at different ages. We then built regression models in which the child's conditional growth measures were entered as a set to predict adult height. Where available we included maternal height, assessed at the time of cohort recruitment, to control for genetic and other intergenerational influences on attained height. We built a series of models to account for differences in data availability across cohorts. We examined the estimates for heterogeneity by gender. Observing only trivial differences between the estimates for men and women that did not affect inference, we present pooled data with adjustment for sex. Adjustment for socioeconomic status at baseline (based on a scale derived from paternal occupation in India and from ownership of household assets in the other cohorts) did not affect the estimates, and results are therefore presented without this adjustment.

## RESULTS

**Fig. 1:**
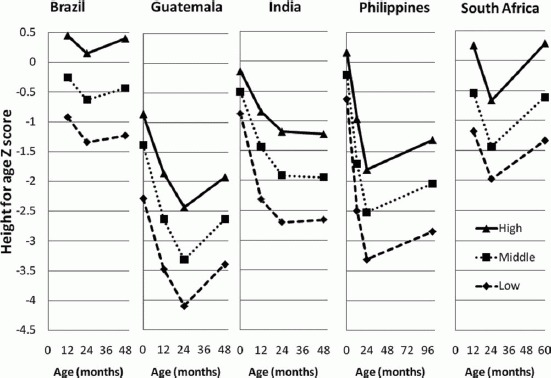
Height for age *Z*-scores at birth, 12 mo, 24 mo, and mid-childhood of participants in five birth cohort studies, stratified by thirds of attained height.

**Fig. 2:**
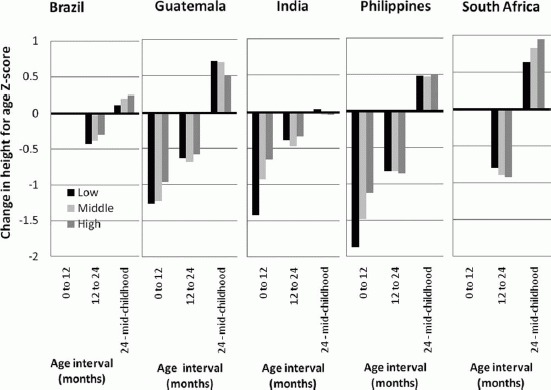
Change in height for age *Z*-score between birth and 12 mo, 12 mo and 24 mo, and 24 mo and mid-childhood among participants in five birth cohort studies, stratified by thirds of attained height.

**Fig. 3:**
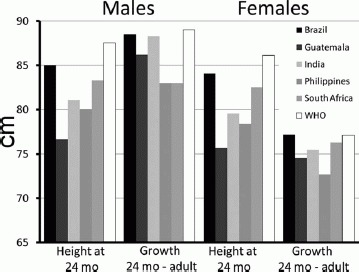
Height at 24 mo and the change in height from age 24 mo through adulthood, by gender, among participants in five birth cohort studies.

The Guatemala and India participants had mean age near 30 years, while the Brazil and Philippines participants were in their early twenties and the South Africa cohort members were adolescents. The five cohorts differed in their growth patterns (Table 2). Mean HAZ at birth ranged from −0.22 (Philippines) to −1.47 (Guatemala). By age of −4 mo, all five cohorts had experienced some degree of growth retardation, with mean HAZ ranging from −0.61 in Brazil to −3.28 in Guatemala. Adult HAZ ranged from −0.35 in Brazil to −1.91 in Guatemala. Figure 1 presents the mean HAZ at specific ages in childhood by cohort, stratified by adult cohort- and sex-specific attained height. Despite the wide range of intercohort mean HAZ, differences across adult height tertiles in HAZ were apparent at birth, and became stable by age of 12 mo. Figure 2 presents these data, expressed as interval change in HAZ. In the three cohorts with birth length, there was a large decrease in HAZ from birth to age 12 mo, with the decrease being larger among those who became shorter adults. Conversely, in the period between 12 and 24 mo, while all cohorts experienced further decreases in mean HAZ, the magnitude of this decrease was not differential by attained height. The extent of increase in HAZ in the period from 24 mo through mid-childhood varied by cohort, being largest in Guatemala and South Africa, and essentially absent in India. There was no consistent relationship across cohorts between the magnitude of the increase in HAZ from 24 mo through mid-childhood and attained height. In all five cohorts, mean height at age 24 mo was 2.0–10.4 cm lower than the WHO reference, whereas growth from age 24 mo through adulthood approximated that of the WHO reference in Brazil, Guatemala, and India (range −0.20 to 2.4 cm) (Fig. 3). For the Philippines cohort, growth in this period was 4.4 cm (females) and 6.0 cm (males) less than the reference, while South African males had not attained adult height at the time of this assessment.

**TABLE 2:** Selected characteristics of participants in five birth cohorts included in present analysis

	Brazil, *N* = 970^a^	Guatemala, *N* = 332	India, *N* = 1,163	Philippines, *N* = 1,873	South Africa, *N* = 321
Maternal height, cm	156.7, 5.9 (958)^b^	148.7, 5.4 (317)		150.5, 4.9	158.6, 6.3 (291)
Gender, % male	50.3	52.7	57.4	53.2	53.0
Age at follow-up, years	15.6, 0.3	32.1, 1.6	29.4, 1.3	21.2,0.9	15.6, 0.3
Length at birth, cm	N/A	49.2, 2.4 (168)	48.6, 2.1 (1021)	49.1,2.0(1872)	N/A
Length at birth, *Z* score^c^	N/A	−1.47, 1.19 (167)	−0.53, 1.09 (1021)	−0.22, 1.08 (1872)	N/A
Length at age 12 mo, cm	73.0, 3.3	68.4, 3.1	71.2, 3.0	70.8, 2.9	73.8, 3.3
Length at age 12 mo, *Z* score	−0.24, 1.07	−2.65, 1.21	−1.72, 1.15	−1.72, 1.13 (1872)	−0.50, 1.28
Length at age 24 mo, cm	84.5, 4.0	780.5, 3.7	80.5, 3.7	79.2, 3.6	79.2, 3.68
Length at age 24 mo, *Z* score	−0.61, 1.14	−3.28, 1.18	−1.94, 1.15	−2.54, 1.10 (1846)	−1.37, 1.17
Height at mid-childhood, cm	100.0, 4.7	92.8, 4.2	94.8, 4.3	93.1,3.9	100.3, 3.8
Height at mid-childhood, *Z* score	−0.43, 1.11	−0.56, 0.97	−1.95, 1.00	−2.39, 0.98	−0.56, 0.97
Males, height at follow-up, cm	173.5, 6.4 (488)	162.9, 6.0 (175)	169.5, 6.3 (668)	163.0, 5.9 (996)	166.3, 7.7 (170)
Females, height at follow-up, cm	161.3, 5.9 (482)	150.3, 6.1 (157)	155.1,5.5(495)	151.1,5.4(877)	158.8, 6.5 (151)
Height at follow-up, *Z* score^3^	−0.35, 0.89	−1.08, 0.87	−1.08, 0.87	−1.85, 0.82	−0.60, 0.97
SGA at birth^d^	15.2 (804)	31.7 (208)	39.6 (949)	24.0 (1835)	16.5 (316)

a Number of valid observations in parentheses if less than full sample.

b Data are mean, S.D., except for gender and SGA, which are presented as percent.

c *Z* scores calculated using WHO 2006; Adult *Z* scores estimated from WHO 2007 at age 19 years.

d SGA, small for gestational age.

The results of the conditional height models are presented in Table 3 for each cohort separately. A full model, incorporating maternal height, birth length, and childhood growth, could be estimated for Guatemala and Philippines. In these models, the coefficients for Guatemala and Philippines are very similar to each other and suggest positive associations between conditional height at any age and adult stature, with the estimates consistent across the two cohorts. In the models with birth length but without maternal height, which now include the India cohort, the conditional height measures are again consistent across cohorts. In the three cohorts, the largest coefficient was for conditional height in the period of 0–12 months. Expanding the number of cohorts by relaxing the requirement for availability of birth length again suggests consistent associations across cohorts. For example, the association of length at 12 mo (which reflects growth from conception through 12 mo) with adult stature ranged from 0.43 (South Africa and Guatemala) to 0.49 (Brazil) and the association for the conditional height at 24 mo ranged from 0.18 (Brazil, India and Philippines) to 0.24 (South Africa). The single notable outlier was the estimate for South Africa for conditional height in mid-childhood, with a coefficient of 0.47 that was approximately twice that of the other four cohorts. The coefficient for conditional height at 24 mo was smaller in all five cohorts than the coefficients for conditional height at other ages.

**TABLE 3:** Site-specific associations between measures of length in childhood and attained height *Z*-score among participants in five birth cohorts

	Brazil		Guatemala		India		Philippines		South Africa	
Models starting from birth										
Cohorts with birth length and maternal height available										
Maternal height *Z* score			0.31	0.20, 0.43			0.22	0.19, 0.25		
Birth length *Z* score			0.27	0.19, 0.35			0.23	0.21, 0.25		
Conditional length at 12 mo			0.31	0.22, 0.39			0.39	0.37, 0.42		
Conditional length at 24 mo			0.12	0.03, 0.20			0.16	0.14, 0.19		
Conditional length at mid-childhood			0.15	0.06, 0.24			0.30	0.27, 0.32		
Cohorts with birth length available										
Birth length *Z* score			0.34	0.26, 0.42	0.22	0.19, 0.26	0.26	0.24, 0.29		
Conditional length at 12 mo			0.35	0.26, 0.44	0.44	0.40, 0.48	0.44	0.41, 0.46		
Conditional length at 24 mo			0.13	0.04, 0.22	0.18	0.14, 0.21	0.18	0.15, 0.20		
Conditional length at mid-childhood			0.18	0.08, 0.27	0.21	0.18, 0.25	0.33	0.30, 0.35		
Models starting from 12 mo										
Cohorts with maternal height available										
Maternal height *Z* score	0.26	0.21, 0.30	0.30	0.21, 0.38			0.22	0.19, 0.26	20.02	20.10, 0.06
Length for age *Z*-score at 12 mo	0.41	0.38, 0.45	0.36	0.31, 0.42			0.40	0.38, 0.42	0.43	0.37, 0.49
Conditional length at 24 mo	0.15	0.11, 0.19	0.17	0.10, 0.23			0.16	0.14, 0.19	0.25	0.18, 0.33
Conditional length at mid-childhood	0.21	0.17, 0.25	0.20	0.14, 0.27			0.30	0.27, 0.32	0.48	0.41, 0.55
All cohorts										
Length for age *Z*-score at 12 mo	0.49	0.45, 0.53	0.43	0.37, 0.48	0.44	0.41, 0.47	0.45	0.43, 0.47	0.43	0.37, 0.48
Conditional length at 24 mo	0.18	0.14, 0.22	0.2	0.13, 0.27	0.18	0.14, 0.22	0.18	0.16, 0.20	0.24	0.17, 0.31
Conditional length at mid-childhood	0.25	0.21, 0.29	0.22	0.16, 0.29	0.22	0.18, 0.25	0.33	0.30, 0.35	0.47	0.41, 0.54

All coefficients are in *Z*-score units of adult height per *Z*-score of the predictor. Blank cells denote models where at least one predictor was not collected and hence model could not be estimated. All models adjusted for age at adult measurement and sex. Estimates and 95% confidence intervals are presented. All estimates *P* < 0.01 except for maternal height in model for South Africa.

The pooled models are presented by data availability in Table 4. The complete model, including maternal height and birth length, could be fitted for the combined Philippines and Guatemala cohorts. This model suggests strong independent associations of conditional height at any period with adult height, with the smallest coefficient observed for 24 mo and the largest for 12 mo. To compare the results for the full model with those for the less constrained models that exclude maternal height or birth length, we fitted reduced models to the combined Philippines and Guatemala samples by successively omitting terms for maternal height and birth length. In both cases, the models differed little from the full model for those parameters included in both sets. Furthermore, the models were stable to the inclusion of additional cohorts. For the models that excluded maternal height, inclusion of the India cohort altered the estimates by only trivial amounts, and in models that included length at 12 mo and conditional heights at 24 mo and mid-childhood, addition of the Brazil and South Africa samples resulted in trivial changes to the estimates.

**TABLE 4:** Pooled analysis of growth in childhood and attained height among participants in five birth cohorts, by type of growth data available

	Guatemala, Philippines		Guatemala, Philippines, India		All five cohorts	
All five parameters included						
Maternal height *Z* score	0.23	0.20, 0.26				
Birth length *Z* score	0.24	0.22, 0.26				
Conditional length at 12 mo^a^	0.38	0.36, 0.41				
Conditional length at 24 mo^b^	0.16	0.14,0.18				
Conditional length at mid-childhood^c^	0.28	0.26, 0.31				
Maternal height not included						
Birth length *Z* score	0.27	0.25, 0.29	0.25	0.24, 0.27		
Conditional length at 12 mo^a^	0.43	0.41, 0.45	0.43	0.41, 0.45		
Conditional length at 24 mo^b^	0.18	0.15,0.20	0.18	0.15, 0.20		
Conditional length at mid-childhood^c^	0.31	0.29, 0.34	0.28	0.26, 0.30		
Maternal height and birth length not included						
Length for age *Z* score at 12 mo	0.45	0.44, 0.47	0.45	0.43, 0.46	0.45	0.44, 0.47
Conditional length at 24 mo^d^	0.18	0.16,0.21	0.18	0.16, 0.20	0.19	0.17, 0.20
Conditional length at mid-childhood^e^	0.31	0.27, 0.30	0.28	0.27, 0.30	0.29	0.27, 0.30

All coefficients are *Z*-score of adult height per *Z*-score unit of the predictor. All models adjusted for site (Philippines as reference), age, and sex. Estimates and 95% confidence intervals are presented.

a Conditional on length at birth.

b Conditional on length at birth and 12 mo.

c Conditional on length at birth, 12 and 24 mo.

d Conditional on length at 12 mo.

e Conditional on length at 12 and 24 mo.

## DISCUSSION

We have analyzed longitudinal data from five birth cohorts in low- and middle-income countries. Despite wide differences in intercohort growth patterns and attained heights, several striking patterns emerged. In all cohorts, there was growth retardation from birth through age 2 years, with modest recovery through mid-childhood.

Growth failure results from the combination of inappropriate feeding practices, including nonexclusive breast feeding through 6 mo, and the increased burden of infection prevalent in lower-resource environments, coupled with suboptimal child care practices (Black et al., 2008). Growth failure is a predictor of poor survival and development of adult human capital in the survivors (Black et al., 2008). Hence, programs to reduce the prevalence of growth failure have both short- and long-term benefits. In a national survey in Brazil, attained height was associated with schooling attainment, occupational choices, employment, and monthly earnings, with the association with earnings being strongest at lower attained heights (Curi and Menenz-Filho, 2008). For men <170 cm in adulthood, each 5 cm increment in height (roughly the increment associated with a 1 SD length deficit at age 12 mo) raised earnings by 250 reais/mo. It is widely presumed that programs to reduce growth failure have the greatest potential for long-term returns if implemented among infants younger than 36 mo (Heckman, 2006). Our data suggest that across these cohorts, growth failure occurs during gestation and in the first 2 years of life, with little additional loss of *Z*-score after that age. Our data also suggest that cohort-specific relative growth failure in the period between 12 and 24 mo is less predictive of adult stature than is growth failure evident at birth or occurring in the first year of life. Data from the Guatemala cohort clearly indicate that provision of food supplements in early childhood (up to age 3 years) reduces the prevalence of stunting (Habicht et al., 1995), and surveillance programs in these same villages have shown that long-term developments in schooling, water supplies, electrifications, and commercial development have coincided with a marked reduction in the prevalence of stunting, from 83% in 1967% to 17% in 2005 (Stein et al., 2009).

The coefficient for the conditional length at age 24 mo was smaller than that for other ages, in all cohorts and in the pooled model. This observation suggests that within-cohort variation in length during the period from 12 mo through 24 mo contributes less to final height than does variation in other periods. This observation is in contrast to the observation that there is a marked growth failure during this same period, as indicated by an overall group-level decline in the HAZ during the second year of life in all five cohorts. The two observations are not mutually exclusive—one is an observation about the group mean, while the other reflects inter-individual variability within the cohort. Furthermore, this is a period of slower overall growth than the period of 0–12 mo, and it is a markedly shorter interval than the period from 24 mo through mid-childhood, which was 24–78 mo in duration. Both these factors may account for the smaller contribution of within-person variability to overall attained height.

Intrauterine life, and the periods between 0 and 24 mo are all periods of growth failure in many settings, including the five cohorts under study here. Because growth failure is cumulative, stunting at 24 mo (or any other period in childhood) represents a very good cumulative (summary) indicator of growth failure during this window of vulnerability. In the Guatemala cohort, the absolute increment in height from 36 mo to adulthood was similar across categories of stunting at 36 mo, whether stunting was considered as being <−3 SD (severe stunting) or <−2.0 SD (stunting) (Martorell et al., 1994). We now show that this observation holds substantively across the five cohorts examined. Taken together with the observation that there was little additional growth failure in the period from 24 mo through mid-childhood, our data further stress the criticality of interventions prior to age 24 mo.

The potential for catch-up growth after age 24 mo may relate to the age at onset of puberty. Thus, even though the annual increment in heights is lower in these populations than in developed countries, the total growth from age 24 mo through adulthood is similar because of delayed puberty. Delayed puberty associated with recovery of height has also been reported in Senegal (Coly et al., 2006). This observation has implications for child and adolescent feeding. As child over-nutrition becomes a more prevalent, nutritional constraints on puberty are removed, and puberty occurs at earlier ages (Ong et al., 2006). It has been suggested that the over-nutrition of the U.S. child and adolescent population is related to the reduction in mean height of the U.S. population, a trend that is not apparent in Dutch adults (Komlos and Breitfelder, 2007). To the extent that is a valid explanation, these data speak to the need to simultaneously address undernutrition in the very young child while preventing over-nutrition in school-age and adolescence.

The five cohorts were established in countries and settings that vary widely. The Guatemala cohort was rural in origin, the Philippines cohort was a mixed rural-urban cohort, and the other three cohorts were urban. The five countries differed in per capita income at the time of cohort inception, and still differ widely. The plane of nutrition and infection differed, as evident by marked differences in the mean growth across cohorts and by the infant mortality rates that varied markedly from 27 (South Africa) to 75 (Guatemala) per thousand (Victora et al., 2008). Nevertheless, the similarities in the derived coefficients are striking, suggesting that the underlying patterns of growth and their relation to adult height are fundamental.

While the large sample size and the range of growth patterns add generalizability to our observations, we were unable in the pooled data to assess the associations between child growth and adult height in finer detail as common data points are lacking. Nevertheless, the intervals we have chosen are relevant for policy as they speak to critical periods in child development, such as the current recommendation to continue breast feeding though age 24 mo (WHO, 2003). The South Africa cohort participants were still growing at the timing of this round of data collection, and hence the data for this cohort are not final. The adult measures were converted to *Z*-scores using a different reference than the child measures, as growth standards are not yet available. Thus, the smaller deficits in height seen in adulthood may reflect this difference rather than any true “catch-up.” However, given the high stability of height ranking from late adolescence through adulthood, the association between growth in childhood and adult stature is unlikely to be markedly affected by the choice of reference. Finally, we were able to use only total height in our analysis and were unable to partition height into trunk or limb components (Gigante et al., 2009).

## CONCLUSIONS

Our data strongly suggest that similarities across these five cohorts outweigh their differences, and that growth failure in childhood, relative to the setting in which the child grows, is a strong determinant of attained height even as the wide difference in mean height across the five cohorts attests to other factors that affect child growth. Efforts to eliminate growth failure in childhood should therefore continue to address both populations with high prevalence of stunting and children at high risk of stunting within settings where the overall prevalence is lower.
